# Comparative Analysis of Anti-Inflammatory Flavones in *Chrysanthemum indicum* Capitula Using Primary Cultured Rat Hepatocytes

**DOI:** 10.3390/molecules30142996

**Published:** 2025-07-16

**Authors:** Keita Minamisaka, Airi Fujii, Cheng Li, Yuto Nishidono, Saki Shirako, Teruhisa Kawamura, Yukinobu Ikeya, Mikio Nishizawa

**Affiliations:** 1 Department of Medical Biosciences, Faculty of Life Sciences, Ritsumeikan University, Kusatsu 525-8577, Shiga, Japanfujii-ai@fc.ritsumei.ac.jp (A.F.); kawater@fc.ritsumei.ac.jp (T.K.); 2 College of Pharmaceutical Sciences, Ritsumeikan University, Kusatsu 525-8577, Shiga, Japan; nisidono@fc.ritsumei.ac.jp; 3 Research Organization of Science and Technology, Ritsumeikan University, Kusatsu 525-8577, Shiga, Japan; y-ikeya@daiichi-cps.ac.jp; 4 Faculty of Pharmacy, Daiichi University of Pharmacy, Fukuoka 815-8511, Fukuoka, Japan; 5 Department of Biology, Faculty of Mathematics and Natural Sciences, Universitas Brawijaya, Malang 65113, East Java, Indonesia

**Keywords:** flavone, 5,7-dihydroxyflavone, inflammation, interleukin 1 receptor, nitric oxide, hepatocyte

## Abstract

The capitula of *Chrysanthemum indicum* Linné or *C. morifolium* Ramatuelle (*Kikuka* in Japanese) are included in several formulae of Kampo medicines (traditional Japanese medicines), such as *Chotosan*, which is used for headache and dizziness. Luteolin, the principal constituent of *C. indicum*, has antioxidant and anti-inflammatory activities. However, the effects of other flavonoids on this crude drug have not yet been thoroughly investigated. To evaluate and compare anti-inflammatory effects, we used primary cultured rat hepatocytes, which produce proinflammatory mediators, such as nitric oxide (NO) and proinflammatory cytokines, in response to interleukin (IL)-1β. Eight derivatives of 5,7-dihydroxyflavone were purified and identified in the ethyl acetate-soluble fraction of a *C. indicum* capitulum extract: luteolin (Compound **1**), apigenin (**2**), diosmetin (**3**), 5,7-dihydroxy-3′,4′,5′-trimethoxyflavone (**4**), acacetin (**5**), eupatilin (**6**), jaceosidin (**7**), and 6-methoxytricin (**8**). Luteolin is the most abundant compound in this fraction. All compounds significantly suppressed NO production in hepatocytes, with apigenin and acacetin showing the greatest efficacy. The comparison of the IC_50_ values of the inhibition of NO production suggests that substitutions by hydroxyl and methoxy groups at the C-3′ and C-4′ positions of 5,7-dihydroxyflavone may be at least essential for the suppression of NO production. In hepatocytes, acacetin and luteolin decreased the levels of mRNAs encoding inducible nitric oxide synthase (iNOS), proinflammatory cytokines, including tumor necrosis factor, IL-6, and type 1 IL-1 receptor, which regulates inflammatory responses. Based on the comparison of the IC_50_ values and the content, luteolin, jaceosidin, and diosmetin may be responsible for the anti-inflammatory effects of *C. indicum* capitula.

## 1. Introduction

*Chrysanthemum* flowers, which are the capitula of *Chrysanthemum indicum* Linné or *C. morifolium* Ramatuelle (Compositae), are used as the crude drug *Kikuka* (in Japanese) in traditional Japanese (Kampo) medicines [1]. *C. indicum* is widely distributed in western Japan and the central and southern parts of China and Taiwan, whereas *C. morifolium* is cultivated throughout Japan and northern China. *C. indicum* has flower heads (3–10 mm in diameter) and ligulate flowers arranged in a single circle that are yellow to light yellow-brown. These flowers have a characteristic odor and a slightly bitter taste [2].

Extracts or compounds from *Chrysanthemum* flowers exhibit antipyretic, detoxifying, antiphlogistic, and anti-inflammatory effects [3]. This crude drug is included in several Kampo formulae, such as *Chotosan* for headache and dizziness and *Kokikujiogan* for feelings of heat and blurred vision. There are flavones (e.g., luteolin, apigenin, and acacetin), terpenoids (e.g., kikkanol and handelin), and phenolic compounds (e.g., caffeic and chlorogenic acids) in the *C. indicum* capitulum [4,5,6]. Flavones possessing a 2-phenylchromen-4-one backbone exhibit anti-inflammatory, antioxidant, and antibacterial activities [7,8].

To date, there are no reports that compare the anti-inflammatory activity of the compounds of the *C. indicum* capitulum using any types of cells. The anti-inflammatory effects of these flavones were evaluated by different assay systems. For example, diosmetin inhibits the dermatitis development of atopic dermatitis model mice [9]. Acacetin protected against sepsis-induced acute lung injury of mice [10].

The proinflammatory mediator nitric oxide (NO) is produced by inducible nitric oxide synthase (iNOS) in hepatocytes and Kupffer cells (i.e., resident macrophages in the liver). In response to bacterial lipopolysaccharide (LPS), Kupffer cells secrete interleukin (IL)-1β, which stimulates hepatocytes to induce NO and proinflammatory cytokines [11]. Primary cultured rat hepatocytes produce NO by iNOS induction in the presence of IL-1β [12,13]. The rat hepatocytes are frequently used as an ex vivo model of liver injury to assess the potential anti-inflammatory activities of compounds, such as in [14]. Excessive NO production is involved in cellular damage during hepatitis-associated liver injury [15].

Luteolin (3′,4′,5,7-tetrahydroxyflavone) is present in medicinal plants, such as the flowers of *C. indicum*, *C. morifolium*, and *C. indicum* var. *albescens* [16]. Luteolin, as well as apigenin (4′,5,7-trihydroxyflavone), inhibited NO production in IL-1β-treated rat hepatocytes [14]. Luteolin inhibits inflammatory responses and protects against vascular inflammation induced by tumor necrosis factor α (TNF-α) [17,18]. In rat hepatocytes, these inflammatory responses are regulated by the IL-1 receptor (IL1R) signaling pathway mediated with the transcription factor nuclear factor κB (NF-κB) [19].

The flavones, excluding luteolin and apigenin, in the *C. indicum* capitula have not been thoroughly investigated, although many flavones and other compounds are included in them [4,5,6]. Furthermore, it is unclear which flavones are responsible for the pharmacological effects, especially the anti-inflammatory effects, of *Chrysanthemum* flowers. In this study, we purified anti-inflammatory flavones from the *C. indicum* capitula by monitoring NO production in IL-1β-treated hepatocytes. We examined whether these flavones suppressed the expression of iNOS and proinflammatory cytokines. Finally, structure–activity relationships of the purified flavones are discussed to elucidate the anti-inflammatory effects of *C. indicum* capitula.

## 2. Results

### 2.1. Extraction of C. indicum Capitula

**Scheme 1:**
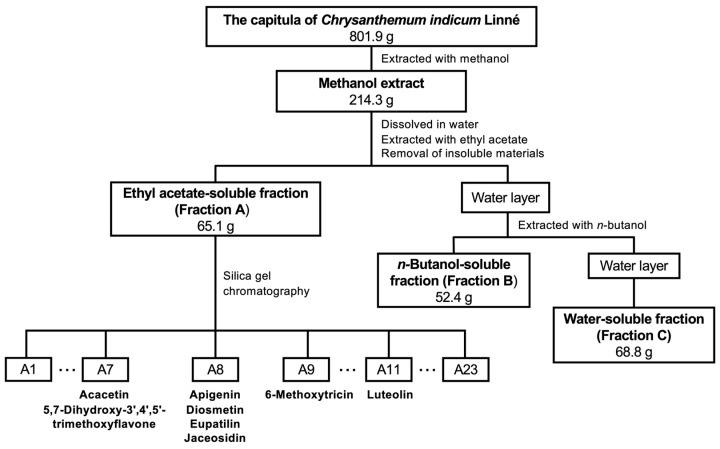
Flowchart of the purification of *C. indicum* capitula. The methanol extract was fractionated into Fractions A, B, and C. Purified compounds are depicted with the relevant subfractions.

The capitula of *C. indicum* were extracted with methanol following previously published methods [20,21], and the resulting extract was fractionated into three crude fractions: ethyl acetate (EtOAc)-soluble Fraction A (Fraction A), the *n*-butanol-soluble fraction (Fraction B), and the water-soluble fraction (Fraction C) (Scheme 1).

**Figure 1:**
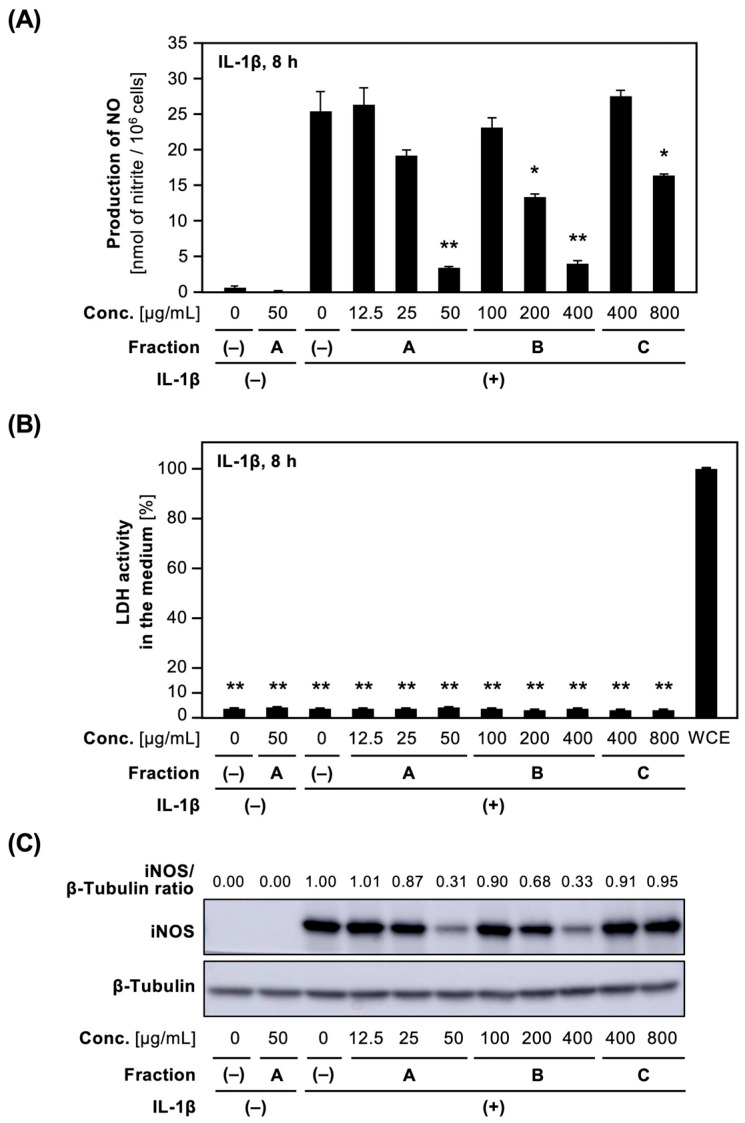
Effect of crude fractions on NO production and iNOS protein expression in hepatocytes. (**A**) Effect of Fractions A, B, and C on NO production. Hepatocytes were treated with 1 nM IL-1β ± Fractions A or B or C for 8 h. The levels of nitrite in the medium are shown as the means ± standard deviations (SDs) (*n* = 3). * *p* < 0.05 and ** *p* < 0.01 versus IL-1β alone. (**B**) The cytotoxic effect of the crude fractions on hepatocytes. The LDH activity in the medium in (**A**) was measured in triplicate. The total LDH activity of the whole-cell extract (WCE) was defined as 100%, and a negative control was Fraction (–). The data represent the means ± SDs (*n* = 3). ** *p* < 0.01 versus Fraction (–). (**C**) Effect of Fractions A, B, and C on iNOS protein expression in hepatocytes. Cell extracts prepared from hepatocytes treated with each crude fraction in (A). The extracts were analyzed by Western blotting to visualize iNOS (130 kDa) and β-tubulin (55 kDa; internal control). The intensity of iNOS band normalized by that of β-tubulin band was shown as an iNOS/β-tubulin ratio above the figure.

The effect of each crude fraction on NO production in IL-1β-treated hepatocytes was examined. The addition of Fractions A and B into the medium decreased IL-1β-induced NO production in a concentration-dependent fashion, whereas Fraction C inhibited NO production at high concentrations of more than 800 μg/mL (Figure 1A). Lactate dehydrogenase (LDH) activity in the medium containing each crude fraction was low compared to that of the whole-cell extract (WCE), i.e., <5% of the WCE (Figure 1B), indicating that none of the fractions at the concentrations applied caused any toxicity to the hepatocytes. Furthermore, Western blot analysis of cell extracts prepared from hepatocytes treated with each crude fraction showed that Fractions A and B decreased iNOS protein expression, whereas Fraction C showed little suppression (Figure 1C).

The IC_50_ values of NO production for the methanol extract and each crude fraction for NO production were determined (Table 1). Fraction A more markedly suppressed NO production in IL-1β-treated hepatocytes compared to Fractions B and C. Fraction A is thought to contain constituents that inhibit NO production in IL-1β-treated hepatocytes.

### 2.2. Identification of Compounds in Fraction A from C. indicum Capitulum (CIC) Extract

To purify the biologically active compounds, Fraction A of the CIC extract was fractionated by silica gel column chromatography to yield subfractions A1–A23, whose ability to suppress NO production was evaluated using hepatocytes. The subfractions that inhibited NO production, A7–A9 and A11, were further purified to obtain eight compounds (Compounds **1**–**8**), as shown in Scheme 1. Their chemical structures were determined by nuclear magnetic resonance (NMR) analysis. ^1^H and ^13^C NMR spectral analyses, heteronuclear multiple quantum correlation spectroscopy, and heteronuclear multiple-bond correlation spectroscopy were also performed.

Compound **1**: yellow powder. ^1^H NMR [500 MHz, (CD_3_)_2_SO, ppm] δ 13.00 (1H, s, 5-OH), 7.43 (1H, dd, *J* = 2.0, 9.0 Hz, H-6′), 7.41 (1H, d, *J* =2.0 Hz, H-2′), 6.99 (1H, d, *J* =9.0 Hz, H-5′), 6.69 (1H, s, H-3), 6.45 (1H, d, *J* = 2.0 Hz, H-8), 6.20 (1H, d, *J* = 2.0 Hz, H-6); and ^13^C NMR [125 MHz, (CD_3_)_2_SO, ppm)] δ 182.1 (C-4), 164.7 (C-7), 164.4 (C-2), 162.0 (C-5), 157.8 (C-9), 150.2 (C-4′), 146.3 (C-3′), 122.0 (C-1′), 119.5 (C-6′), 116.5 (C-5′), 113.9 (C-2′), 104.2 (C-10), 103.4 (C-3), 99.4 (C-6), and 94.4 (C-8). This compound was identified as luteolin based on ^1^H and ^13^C NMR spectral analysis and comparison with previously published results [14,22].

Compound **2**: yellow powder. ^1^H NMR (500 MHz, CD_3_OD, ppm) δ 7.84 (2H, d, *J* = 9.0 Hz, H-2′, H-6′), 6.92 (2H, d, *J* = 9.0 Hz, H-3′, H-5′), 6.57 (1H, s, H-3), 6.42 (1H, d, *J* = 2.0 Hz, H-8), 6.17 (1H, d, *J* = 2.0 Hz, H-6); and ^13^C NMR (125 MHz, CD_3_OD, ppm) δ 183.7 (C-4), 167.6 (C-7), 166.1 (C-2), 163.1 (C-5), 162.9 (C-4′), 159.5 (C-9), 129.4 (C-2′, C-6′), 123.1 (C-1′), 117.0 (C-3′, C-5′), 104.8 (C-10), 103.6 (C-3), 100.6 (C-6), and 95.4 (C-8). This compound was identified as apigenin based on ^1^H and ^13^C NMR spectral analyses and comparison with previously published results [14,23].

Compound **3**: yellow powder. ^1^H NMR [500 MHz, (CD_3_)_2_SO, ppm] δ 7.52 (1H, dd, *J* = 2.0, 9.0 Hz, H-6′), 7.42 (1H, d, *J* =2.0 Hz, H-2′), 7.08 (1H, d, *J* =9.0 Hz, H-5′), 6.70 (1H, s, H-3), 6.39 (1H, s, H-8), 6.12 (1H, s, H-6), 3.86 (3H, s, 4′-OCH_3_); and ^13^C NMR [125 MHz, (CD_3_)_2_SO, ppm] δ 181.2 (C-4), 166.4 (C-7), 163.0 (C-2), 161.3 (C-5), 157.4 (C-9), 150.9 (C-4′), 146.7 (C-3′), 123.0 (C-1′), 118.4 (C-6′), 112.7 (C-2′), 112.0 (C-5′), 103.2 (C-10), 102.8 (C-3), 99.3 (C-6), 94.1 (C-8), and 55.6 (4′-OCH_3_). This compound was identified as diosmetin based on ^1^H and ^13^C NMR spectral analyses and comparison with previously published results [24].

Compound **4**: yellow powder. ^1^H NMR [500 MHz, (CD_3_)_2_SO, ppm] δ 7.34 (2H, s, H-2′, H-6′), 7.08 (1H, s, H-3), 6.56 (1H, s, H-8), 6.20 (1H, s, H-6), 3.90 (6H, s, 3′-OCH_3_, 5′-OCH_3_), 3.75 (3H, s, 4′-OCH_3_); and ^13^C NMR [125 MHz, (CD_3_)_2_SO, ppm] δ 181.8 (C-4), 164.8 (C-7), 162.9 (C-2), 161.3 (C-5), 157.4 (C-9), 153.2 (C-3′, C-5′), 140.6 (C-4′), 126.0 (C-1′), 104.9 (C-3), 104.0 (C-2′, C-6′), 103.6 (C-10), 99.1 (C-6), 94.4 (C-8), 60.2 (4′-OCH_3_), and 56.2 (3′-OCH_3_, 5′-OCH_3_). This compound was identified as 5,7-dihydroxy-3′,4′,5′-trimethoxyflavone based on ^1^H NMR and ^13^C NMR spectra analysis and comparison with previously published results [25]. This is the first time that Compound **4** has been identified in a CIC extract.

Compound **5**: yellow powder. ^1^H NMR [500 MHz, (CD_3_)_2_SO, ppm] δ 8.05 (2H, d, *J* = 9.0 Hz, H-2′, H-6′), 7.12 (2H, d, *J* =9.0 Hz, H-3′,H-5′), 6.88 (1H, s, H-3), 6.50 (1H, d, *J* =2.0 Hz, H-8), 6.19 (1H, d, *J* =2.0 Hz, H-6), 3.86 (3H, s, 4′-OCH_3_); and ^13^C NMR [125 MHz, (CD_3_)_2_SO, ppm] δ 181.6 (C-4), 164.4 (C-7), 163.1 (C-2), 162.2 (C-4′), 161.3 (C-5), 157.2 (C-9), 128.2 (C-2′, C-6′), 122.7 (C-1′), 114.5 (C-3′,C-5′), 103.5 (C-10), 103.4 (C-3), 98.8 (C-6), 94.0 (C-8), and 55.5 (4′-OCH_3_). This compound was identified as acacetin based on ^1^H and ^13^C NMR spectral analyses and comparison with previously published results [24].

Compound **6**: yellow powder. ^1^H NMR (500 MHz, CD_3_OD, ppm) δ 7.56 (1H, dd, *J* = 2.0, 9.0 Hz, H-6′), 7.43 (1H, d, *J* =2.0 Hz, H-2′), 7.07 (1H, d, *J* = 9.0 Hz, H-5′), 6.57 (1H, s, H-3), 6.44 (1H, s, H-8), 3.92 (3H, s, 3′-OCH_3_), 3.90 (3H, s, 4′-OCH_3_), 3.85 (3H, s, 6-OCH_3_); and ^13^C NMR (125 MHz, CD_3_OD, ppm) δ 183.6 (C-4), 165.1 (C-2), 163.9 (C-2), 155.3 (C-9), 153.8 (C-4′), 153.7 (C-5), 150.8 (C-3′), 134.2 (C-6), 125.2 (C-1′), 121.3 (C-6′), 112.7 (C-5′), 110.3 (C-2′), 104.2 (C-10), 103.9 (C-3), 96.6 (C-8), 60.7 (C-6), 60.7 (6-OCH_3_), 56.7 (3′-OCH_3_), and 56.5 (4′-OCH_3_). This compound was identified as eupatilin based on ^1^H and ^13^C NMR spectral analyses and comparison with previously published results [26].

Compound **7**: yellow powder. ^1^H NMR (500 MHz, CD_3_OD, ppm) δ 7.47 (1H, dd, *J* = 2.0, 9.0 Hz, H-6′), 7.44 (1H, d, *J* =2.0 Hz, H-2′), 6.90 (1H, d, *J* = 9.0 Hz, H-5′), 6.51 (1H, s, H-3), 6.39 (1H, s, H-8), 3.95 (3H, s, 3′-OCH_3_), 3.84 (3H, s, 6-OCH_3_); and ^13^C-NMR (125 MHz, CD_3_OD, ppm) δ 183.4 (C-4), 166.2 (C-7), 165.4 (C-2), 155.6 (C-9), 153.6 (C-5), 152.5 (C-4′), 149.7 (C-3′), 134.8 (C-6), 123.7 (C-1′), 121.5 (C-6′), 116.9 (C-5′), 110.3 (C-2′), 103.4 (C-10), 103.1 (C-3), 97.2 (C-6), 60.6 (6-OCH_3_), and 56.6 (3′-OCH_3_). This compound was identified as jaceosidin based on ^1^H and ^13^C NMR spectral analyses and comparison with previously published results [26].

Compound **8**: yellow powder. ^1^H NMR [500 MHz, (CD_3_)_2_SO, ppm] δ 7.22 (2H, s, H-2′, H-6′), 6.73 (1H, s, H-3), 6.31 (1H, s, H-8), 3.85 (6H, s, 3′-OCH_3_, 5′-OCH_3_), 3.71 (3H, s, 6-OCH_3_); and ^13^C-NMR [125 MHz, (CD_3_)_2_SO, ppm] δ 180.5 (C-4), 162.0 (C-2), 156.0 (C-7), 153.4 (C-9), 151.9 (C-5), 148.2 (C-3′, C-5′), 141.3 (C-4′), 132.8 (C-6), 128.6 (C-1′), 103.8 (C-2′, C-6′), 102.2 (C-3), 100.7 (C-10), 95.4 (C-8), 59.1 (6-OCH_3_), and 56.1 (3′-OCH_3_, 5′-OCH_3_). This compound was identified as 6-methoxytricin based on ^1^H and ^13^C NMR spectral analysis and comparison with previously published results [27].

**Figure 2:**
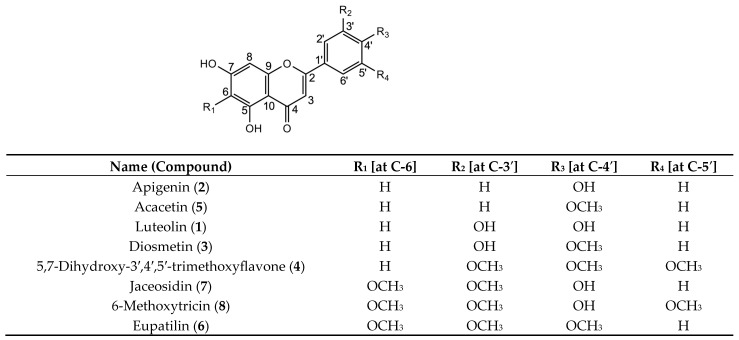
Chemical structures of derivatives of 5,7-dihydroxyflavone purified from Fraction A of the *C. indicum* capitulum (CIC) extract.

The above-mentioned ^1^H and ^13^C NMR signals were assigned, and the chemical structures were determined. All identified compounds were derivatives of 5,7-dihydroxyflavones (Figure 2).

### 2.3. Flavone Content in Fraction A of the CIC Extract

**Figure 3:**
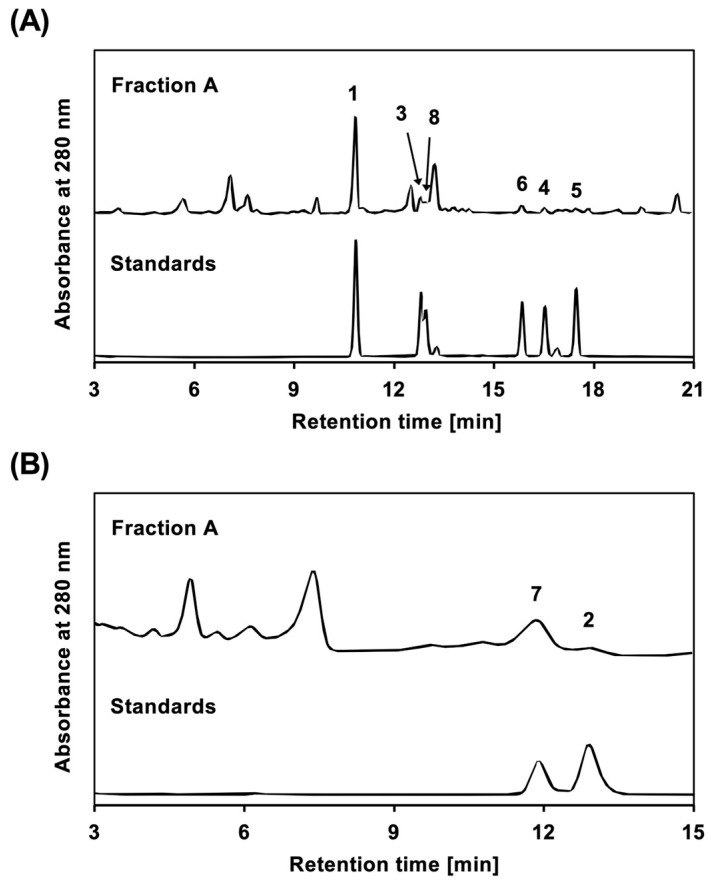
Compounds present in CIC Fraction A. HPLC chromatograms are shown for CIC Fraction A and standards in (**A**,**B**). Compounds **1** to **8** were used as standards. Fraction A and the standards were analyzed by HPLC as described in Materials and Methods. The number on each peak of the Fraction A chromatograms corresponds to the compound number.

The content of each 5,7-dihydroxyflavone derivative in CIC Fraction A was examined. As shown in Figure 3, eight compounds were analyzed using HPLC, and the content of each compound was calculated.

Among the compounds isolated from Fraction A, luteolin (**1**) was the most abundant (Table 2). The effects of the purified compounds on hepatocytes were examined using an NO assay, as described in Materials and Methods. The IC_50_ values of the inhibition of NO production were determined for three different concentrations of each compound unless it showed cytotoxicity, and at least three independent experiments were performed to confirm reproducibility. When the IC_50_ values of luteolin and apigenin (**2**) were determined in the current study, they were comparable to those that we previously reported using primary cultured rat hepatocytes [14]. Additionally, the inhibitory effects of chrysin, a 5,7-dihydroxyflavone, have also been examined. Chrysin is mostly found in food sources, including green vegetables [28]. The IC_50_ values of the flavones are summarized in Table 2. These results suggested that apigenin and acacetin (**5**) effectively inhibited NO production.

The contribution of a compound in Fraction A inhibition of NO production was correlated with its content and inversely correlated with its IC_50_ value. Therefore, it was represented as the content/IC_50_ ratio. This calculation suggests that luteolin, jaceosidin, and diosmetin contribute to the inhibition of NO production. Although the eight derivatives of 5,7-dihydroxyflavone were identified by monitoring the inhibition of NO production using hepatocytes, the sum of the content of all the compounds was only 2.87% in Fraction A.

### 2.4. Effects of CIC Flavones on NO Production in IL-1β-Treated Hepatocytes

**Figure 4:**
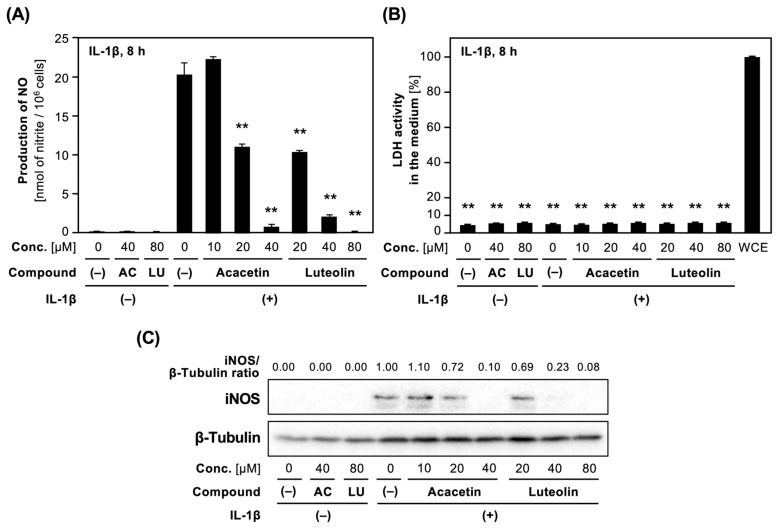
Effects of acacetin and luteolin on NO production and iNOS protein expression in hepatocytes. (**A**) Effect on NO production. Hepatocytes were treated with 1 nM IL-1β ± luteolin (LU) or acacetin (AC) for 8 h. The levels of nitrite in the medium were measured and are shown as the means ± SDs (*n* = 3). ** *p* < 0.01 versus IL-1β alone. (**B**) The cytotoxic effect of acacetin and luteolin on hepatocytes. The LDH activity in the medium in (**A**) was measured in triplicate. The total LDH activity represents the mean ± SD (*n* = 3). ** *p* < 0.01 versus Fraction (–). (**C**) Effect on iNOS protein expression. Cell extracts prepared from hepatocytes treated with each compound. The extracts were analyzed by Western blotting to detect and visualize iNOS and β-tubulin (internal control). The intensity of iNOS band normalized by that of β-tubulin band is shown as an iNOS/β-tubulin ratio above the figure.

Next, we examined whether the derivatives of 5,7-dihydroxyflavone suppress the expression of iNOS protein in IL-1β-treated hepatocytes. Acacetin (**5**), as well as apigenin (**2**), possessed the lowest IC_50_ values among the compounds with newly determined IC_50_ values (Table 2). Acacetin (**5**) and luteolin (**1**) decreased IL-1β-induced NO production in a concentration-dependent manner (Figure 4). Because the LDH activity in the medium containing each compound was low, the compounds were not toxic at the concentrations applied. Western blot analysis indicated that acacetin and luteolin decreased IL-1β-induced expression of iNOS protein in hepatocytes.

### 2.5. Effects of CIC Flavones on the Expression of the iNOS mRNA in Hepatocytes

We examined whether the active flavones isolated from the CIC extract affected the mRNA expression of proinflammatory genes. Two typical compounds, acacetin (with the high activity for suppressing NO production) and luteolin (a major constituent), were selected. After the incubation of hepatocytes with each compound and IL-1β, the mRNA levels were measured by quantitative reverse transcription–polymerase chain reaction (RT–qPCR). The expression level of each mRNA was normalized to the expression level of the internal control elongation factor 1α (*Ef1a*) mRNA [13].

**Figure 5:**
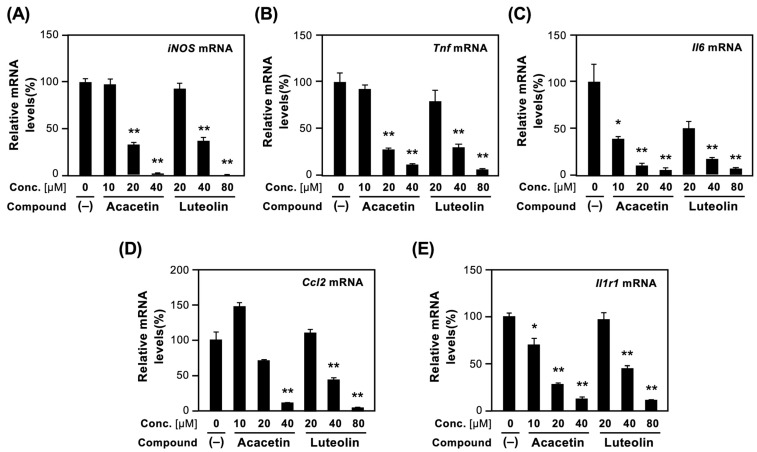
Effects of acacetin and luteolin on the mRNA expression of proinflammatory genes. After incubation of hepatocytes treated with each compound and IL-1β for 4 h, total RNA was prepared, and mRNA levels were measured by RT–qPCR. The levels of each mRNA were measured in triplicate and normalized to the *Ef1a* mRNA levels: (**A**) *iNOS* mRNA; (**B**) *Tnf* mRNA; (**C**) *Il6* mRNA; (**D**) *Ccl2* mRNA; and (**E**) *Il1r1* mRNA. Relative mRNA levels (%) are shown as the means ± SDs (*n* = 3). In the presence of IL-1β alone, the mRNA level measured was set as 100%. When IL-1β was not added or when each compound alone was added to the medium, amplification was not detected. * *p* < 0.05 and ** *p* < 0.01 versus IL-1β alone.

As shown in Figure 5A, the *iNOS* mRNA was induced by IL-1β. When luteolin was added to the culture medium, *iNOS* mRNA levels decreased in a concentration-dependent manner. Acacetin more efficiently reduced the *iNOS* mRNA levels than luteolin did.

### 2.6. Effects of CIC Flavones on Proinflammatory Gene Expression in Hepatocytes

Similarly, the mRNA expression of proinflammatory cytokines, such as TNF-α (*Tnf*) and IL-6 (*Il6*), and chemokine (C-C motif) ligand 2 (*Ccl2*), decreased in hepatocytes. Luteolin and acacetin reduced *Tnf*, *Il6*, and *Ccl2* mRNA levels in the presence of IL-1β (Figure 5B–D).

Lastly, the expression of type 1 IL-1 receptor (*Il1r1*) mRNA was examined because IL1R1 is a key molecule involved in proinflammatory responses. As shown in Figure 5E, luteolin and acacetin decreased *Il1r1* mRNA levels in a concentration-dependent manner. Collectively, these results indicated that luteolin and acacetin possess anti-inflammatory effects.

## 3. Discussion

The eight compounds purified from Fraction A of the CIC extract were derivatives of 5,7-dihydroxyflavone. Among them, 5,7-dihydroxy-3′,4′,5′-trimethoxyflavone (**4**) was isolated for the first time from the capitulum of *C. indicum* in this study. These compounds effectively inhibited NO production in hepatocytes. The contribution of a compound to the inhibition of NO production was represented as a content/IC_50_ ratio, and luteolin was found to contribute the most (Table 2). Although we identified the eight derivatives of 5,7-dihydroxyflavone by monitoring the inhibition of NO production using hepatocytes, the sum of the content of all the compounds was only 2.87% in CIC Fraction A. When compared to the IC_50_ value of Fraction A (32.8 μg/mL), other compounds, such as terpenoids and phenolic compounds, may be involved in the inhibition of NO production. Although chlorogenic acid also suppressed NO production in primary cultured rat hepatocytes, it is included in Fraction C, not Fraction A [20]. Kikkanols suppressed NO production in LPS-activated mouse macrophages [5]. Therefore, these terpenoids should be examined in the future.

The derivatives of 5,7-dihydroxyflavone in the CIC extract exhibited anti-inflammatory effects by downregulating the *Il1r1* gene (Figure 5). IL-1β plays a central role in inflammatory responses via its receptors, including IL1R1 [19,29,30]. Luteolin and acacetin decreased the expression of iNOS and proinflammatory cytokines (e.g., IL-6, TNF-α, and CCL2), leading to the decreases in proinflammatory mediators, such as NO (Figure 4). These results are in accordance with the previous data that luteolin reduced NO production and the expression of *iNOS* and *Tnf* mRNAs [14].

Except for this study, there are no reports that compare the anti-inflammatory activity of the 5,7-dihydroxyflavone derivatives of the *C. indicum* capitulum using one assay system. Anti-inflammatory effects were reported by different assay systems. Diosmetin inhibits the dermatitis development of atopic dermatitis model mice [9]; 5,7-dihydroxy-3′,4′,5′-trimethoxyflavone possessed neuroprotective effects against lead-induced brain toxicity in rats [31]; and acacetin protected against sepsis-induced acute lung injury of mice [10]. Furthermore, eupatilin suppressed iNOS and proinflammatory cytokines in LPS-treated macrophage line J774A.1 [32]; jaceosidin isolated from *Artemisia princeps* Pampanini *cv*. Sajabal suppressed the iNOS expression in LPS-treated macrophage line RAW264.7 [33]; and 6-methoxytricin isolated from *Artemisia vestita* inhibited the proliferation and activation of T cells in vitro [34].

To discuss the structure–activity relationship, the compounds examined in this study were aligned with the IC_50_ values using IL-1β-treated rat hepatocytes (Table 2). It is essential to compare IC_50_ values of the compounds using the same assay system. Substitution of hydrogen by the hydroxyl group caused increases in the IC_50_ values, which was seen in the cases of apigenin and luteolin at the C-3′ position and acacetin and diosmetin at the C-3′ position. These data reflect previously reported findings that the IC_50_ value of luteolin (20 μM) was higher than that of apigenin (7.7 μM) in LPS-treated macrophage line RAW 264.7 [35].

Substitution of hydrogen by a methoxy group increased the IC_50_ values, which were observed for chrysin (5,7-dihydroxyflavone) and eupatilin (C-6, C-3′, and C-4′ positions), and chrysin (5,7-dihydroxyflavone) and 5,7-dihydroxy-3′,4′,5′-trimethoxyflavone (C-3′, C-4′, and C-5′ positions). In addition, the hydroxyl group was substituted by the methoxy group at the C-4′ position and decreased the IC_50_ values, which was seen in the cases of luteolin and diosmetin, and jaceosidin and eupatilin. The former case is in accordance with a previous report that an IC_50_ value of luteolin (20 μM) was higher than that of diosmetin (8.9 μM) in LPS-treated RAW 264.7 cells [35]. Collectively, the substitutions at the C-3′ and C-4′ positions seem to be essential for the suppression of NO production.

Furthermore, the C-6 substituent may have suppressed NO production. There are more derivatives of 5,7-dihydroxyflavone, including substitution at the C-6 position, e.g., baicalein (= 5,6,7-trihydroxyflavone) [36] and nepetin (= 3′,4′,5,7-tetrahydroxy-6-methoxyflavone) [37]. Further studies are required to elucidate the effects of substitution (e.g., hydroxylation and methylation) on the activity of the 5,7-dihydroxyflavone derivatives.

CIC is used in several Kampo formulae, such as *Chotosan* and *Kokikujiogan*, for headache, feeling of heat, and blurred vision. The anti-inflammatory properties of the 5,7-Dihydroxyflavone derivatives may explain the usage of these Kampo formulae. In addition, *Chotosan* ameliorates cognitive performance in animal models of dementia and has potential usefulness in dementia patients [38]. Although the 5,7-dihydroxyflavone derivatives are expected to be partly responsible for the beneficial effects on dementia, there is a lack of data on the mechanisms of actions of key constituents. Further investigation using in vivo models is necessary to examine the involvement of the 5,7-dihydroxyflavone derivatives as well as the pharmacological effects of Kampo formulae, including *Chotosan* and *Kokikujiogan*.

## 4. Materials and Methods

### 4.1. General Experimental Procedures

Column chromatography was performed using Silica Gel 60 (Nacalai Tesque, Inc., Kyoto, Japan), Wakogel C-300 HG (Fujifilm Wako Pure Chemical Corporation, Osaka, Japan), and Sephadex LH-20 (Sigma-Aldrich, St. Louis, MO, USA). Analytical and preparative thin-layer chromatography (TLC) was performed using Silica gel 60 F_254_ plates (Fujifilm Wako Pure Chemical Corporation). Preparative TLC was performed on Silica gel 70 PF_254_ plates (Fujifilm Wako Pure Chemical Corporation) or PLC Silica gel 60 F_254_ (Merck KGaA, Darmstadt, Germany). Chrysin was purchased from Fujifilm Wako Pure Chemical Corporation. NMR spectra were recorded using a JNM-ECZ500R NMR spectrometer (JEOL Ltd., Akishima, Tokyo, Japan) operated at 500 MHz (^1^H) and 125 MHz (^13^C). Tetramethylsilane (internal standard), deuterated methanol (CD_3_OD), and dimethyl sulfoxide [(CD_3_)_2_SO] were obtained from Euriso Top (Saint-Aubin, France). Chrysin was purchased from Fujifilm Wako Pure Chemical Corporation.

### 4.2. Plant Material

*C. indicum* capitula collected from the Guangxi Zhuang Autonomous Region, China, and authenticated by Dr. Yutaka Yamamoto (Tochimoto Tenkaido Co., Ltd., Osaka, Japan) were obtained from Tochimoto Tenkaido Co., Ltd. A voucher specimen was deposited in the Ritsumeikan Herbarium of Pharmacognosy, Ritsumeikan University (No. RIN-CI-39).

### 4.3. Extraction and Fractionation of the Methanol Extract

The capitula of *C. indicum* (801.9 g) were pulverized and extracted with methanol (5 L) under reflux three times and evaporated in vacuo, yielding 214.3 g (26.7%), according to the previously established method [20]. The resulting CIC extract was dissolved in water and extracted by sequential partitioning with EtOAc and *n*-butanol into Fractions A, B, and C (Figure 1). Fraction A, which showed the highest inhibitory activity against NO production in hepatocytes, was purified from these compounds.

### 4.4. Isolation of the Compounds from CIC Extract

Fraction A (65.1 g) of the CIC extract was subjected to silica gel column chromatography (7.0 cm internal diameter × 30 cm), and the compounds were eluted stepwise using *n*-hexane:acetone (100:0, 90:10, 80:20, 70:30, 60:40, 50:50, 40:60, and 0:100) to obtain subfractions A1 to A23 by monitoring eluted compounds on TLC. The yield of the subfractions was 94.5% of that of the starting material. These subfractions were subjected to NO assays, and subfractions that showed high inhibitory activities against NO production were purified to obtain compounds. Subfraction A11 was recrystallized from chloroform, and the resultant yellow powder was designated as Compound **1** (187.5 mg). Subfraction A8 (2.01 g) was subjected to silica gel column chromatography using *n*-hexane:acetone (80:20–0:100) to obtain subfractions A8-1 to A8-12. Subfraction A8-7 (60 mg) was purified via preparative TLC using chloroform:methanol (8:1) to obtain Compounds **2** (3.23 mg) and **3** (6.35 mg). Subfraction A7 (1.55 g) was subjected to silica gel column chromatography using chloroform:methanol (100:0–0:100) to obtain subfractions A7-1 to A7-9. Subfraction A7-4 (198 mg) was purified by preparative HPLC to obtain Compounds **4** (3.80 mg) and **5** (4.60 mg). Subfractions A8-4 (30 mg) and A8-6 (30 mg) were purified by preparative TLC using chloroform:methanol:water (10:1:0.1) to obtain Compounds **6** (6.39 mg) and **7** (4.73 mg), respectively. Subfraction A9 (600 mg) was subjected to column chromatography using silica gel and then Sephadex LH-20 (Cytiva, Marlborough, MA, USA) using water:methanol (50:50) and (0:100) to obtain A9-1 and A9-2, respectively. Subfraction A9-2 (48.9 mg) was purified by preparative TLC using chloroform:methanol (12:1) to obtain Compound **8** (11.3 mg). The compounds were subjected to NMR spectroscopy and NO assays.

### 4.5. Measuring Compound Content Using HPLC

According to a previously published method [21], the content was measured by HPLC using an LC-20AT pump, an SPD-20A UV/VIS detector (Shimadzu Corporation, Kyoto, Japan), and a Cosmosil 5C_18_ MS-II column or Cosmosil 5C_18_ AR-II column (4.6 mm internal diameter × 150 mm; Nacalai Tesque, Inc.). The compounds were eluted at a flow rate of 1.0 mL/min using a linear gradient (0–20 min) of a mobile phase consisting of 20 mM phosphate buffer (pH 2.5) and an acetonitrile mixture (80:20 to 30:70) with a Cosmosil 5C_18_ MS-II column and detected at a wavelength of 280 nm (Figure 3). Single peaks with retention times of 10.9, 12.8, 13.1, 15.9, 16.5, and 17.7 min corresponded to Compounds **1**, **3**, **8**, **6**, **4**, and **5**, respectively. The compounds were eluted at a flow rate of 1.0 mL/min using an isocratic mobile phase of 0.1% formic acid in a water:methanol mixture (45:55) on a Cosmosil 5C_18_ AR-II column and detected at a wavelength of 280 nm. Single peaks with retention times of 11.7 min and 12.2 min corresponded to Compounds **7** and **2**. Each purified compound was used as a standard after accurate weighing and dissolution in methanol to 1.00 mg/mL. Fraction A was prepared at concentrations of 5.00 mg/mL or 10.0 mg/mL. Each solution was further diluted to prepare a standard solution. The content of each standard compound in Fraction A was calculated based on a calibration curve: Compound **1**, *y* = 36,232,070 *x* − 116,451.8 (*R*^2^ = 0.9999); **3**, *y* = 15,988,490 *x* − 25,184.19 (*R*^2^ = 0.9919); **8**, *y* = 14,411,420 *x* − 20,818.73 (*R*^2^ = 0.9942); **6**, *y* = 14,411,420 *x* − 20,818.73 (*R*^2^ = 0.9942); **4**, *y* = 13,819,040 *x* − 22,135.54 (*R*^2^= 0.9914); **5**, *y* = 15,153,310 *x* − 21,647.23 (*R*^2^ = 0.9943); **7**, *y* = 16,682,390 *x* + 11,745.68 (*R*^2^ = 0.9969); and **2**, *y* = 17,484,190 *x* − 21,263.43 (*R*^2^ = 0.9950). The contents were 0.80%, 0.32%, 0.16%, 0.27%, 0.16%, 0.27%, 0.77%, and 0.13%, respectively.

### 4.6. Animals and Primary Cultured Rat Hepatocytes

All animal care and experimental procedures were performed in accordance with the laws and guidelines of the Japanese government and were approved by the Animal Care Committee of Ritsumeikan University, Biwako-Kusatsu Campus (Nos. BKC2020-045 and BKC2023-052). Wistar rats were acclimatized, and hepatocytes were prepared according to previously reported methods [21]. Hepatocytes were seeded at 1.2 × 10^6^ cells per 35 mm diameter dish, and the medium was replaced with serum-free Willams’ E (WE) medium after seeding. The cells were incubated overnight at 37 °C, and the medium was replaced with WE medium containing 1 nM recombinant rat IL-1β and/or fraction or compound.

### 4.7. NO Assay and LDH Activity

The cells were incubated in WE medium containing 1 nM IL-1β ± fraction or compound for 8 h at 37 °C, and nitrite, a stable metabolite of NO, was measured by the Griess method of previously reported methods [12,39]. The IC_50_ values of inhibition of NO production were determined for three different concentrations of a fraction or a compound unless it showed cytotoxicity, as previously described [40]. Each IC_50_ value (mean ± SD) was calculated by at least three independent experiments using hepatocytes to confirm reproducibility. Hepatocyte cytotoxicity was assessed by estimating LDH activity in the medium using an LDH Cytotoxicity Detection Kit (Takara Bio Inc., Kusatsu, Japan) or an LDH-Glo Cytotoxicity Assay Kit (Promega Corporation, Madison, WI, USA). The LDH activity of the whole-cell extracts was assumed to be 100%. When the LDH activity in the medium containing a compound was low (i.e., less than about 5% of the whole-cell extract), this compound was assumed to be non-toxic to hepatocytes.

### 4.8. Western Blot Analysis and Densitometry

Western blotting was performed using a previously described method [21]. Briefly, hepatocytes were incubated with 1 nM IL-1β ± fraction or compound for 8 h. Cell lysates were prepared in the presence of a protease inhibitor cocktail (Nacalai Tesque, Inc.). The resulting lysates were electrophoresed on a 10% sodium dodecyl sulfate–polyacrylamide gel and transferred onto a Sequi-Blot membrane (Bio-Rad, Hercules, CA, USA). After blocking with 5% skim milk, the target proteins were immunostained with primary antibodies against iNOS (BD Biosciences, Franklin Lakes, NJ, USA) and β-tubulin (Cell Signaling Technology Inc., Danvers, MA, USA), followed by incubation with horseradish peroxidase-conjugated anti-immunoglobulin Fc antibody. The proteins were visualized using ECL Blotting Detection Reagents (Cytiva) and detected using an Amersham Imager 600 (Cytiva). To measure intensity of iNOS bands, densitometry was performed using ImageJ software Version 1.54p (https://imagej.net/ij/, accessed on 17 February 2025). The band intensity was normalized by that of a β-tubulin (control) band. The ratio of the band intensity (iNOS/β-tubulin ratio) was calculated.

### 4.9. RT–qPCR

Hepatocytes were incubated with 1 nM IL-1β ± fraction or compound for 4 h; total RNA was prepared from hepatocytes using Sepasol I Super G solution (Nacalai Tesque, Inc.) according to a previously published method [13]. cDNA was synthesized from total RNA and amplified by PCR using specific primers [21]. Real-time PCR was performed using SYBR Green I and the Thermal Cycler Dice Real Time System (Takara Bio Inc.), and mRNA levels were quantitatively measured in triplicate. We confirmed the presence of a single melting temperature peak in the dissociation curve of the amplified product for each mRNA. Relative mRNA levels were calculated from the obtained threshold cycle (Ct) values according to the ΔΔCt method. The Ct values were normalized to *Ef1a* mRNA [13], a housekeeping gene (an internal control). The normalized mRNA levels in the total RNA from IL-1β-treated hepatocytes were set to 100%.

### 4.10. Statistical Analysis

The data are representative of at least three independent experiments with similar findings. Each value is presented as a mean ± standard deviation (SD), and the differences were analyzed using the Student’s *t*-test with Bonferroni correction. The significance was set at *p* < 0.05 and *p* < 0.01.

## 5. Conclusions

Guided by the suppression of NO production in IL-1β-treated hepatocytes, we identified eight 5,7-dihydroxyflavone derivatives of CIC extract. These flavones inhibit NO production and iNOS expression. Based on the comparison of the content and IC_50_ values of the inhibition of NO production, luteolin, jaceosidin, and diosmetin may be responsible. Luteolin and acacetin downregulated the expression of proinflammatory genes encoding iNOS and cytokines. These 5,7-dihydroxyflavone derivatives may contribute to the pharmacological effects, especially anti-inflammatory effects, of the capitula of *C. indicum*. This study will facilitate the investigation of Kampo formulae, such as *Chotosan* and *Kokikujiogan*.

## Figures and Tables

**Table 1:** Effects of crude fractions of *C. indicum* capitula on NO production in hepatocytes.

Extract/Crude Fraction	Yield [%] ^1^	IC_50_ [µg/mL] ^2^
Methanol extract	100	75.6 ± 11.9
Fraction A (EtOAc-soluble)	31.5	32.8 ± 3.09
Fraction B (*n*-butanol-soluble)	17.1	210 ± 22.2
Fraction C (water-soluble)	51.4	NA

^1^ The percentage of weight was calculated by dividing each fraction by the sum of the three fractions. ^2^ Each value (mean ± SD) was calculated by at least three independent experiments. NA, not applied because of low activity.

**Table 2:** Effects of the derivatives of 5,7-dihydroxyflavone on NO production in hepatocytes.

Name (Compound)	Content in the CIC Fraction A [%] ^1^	IC_50_ [µM] ^2^	Content/IC_50_ Ratio	Content/IC_50_ [%] ^3^
Chrysin (5,7-dihydroxyflavone)	ND	11.6 ± 0.56	–––	–––
Apigenin (**2**)	0.13	12 ± 5.4 ^4^	0.007	26.9
Acacetin (**5**)	0.16	14.6 ± 1.73	0.011	41.9
Luteolin (**1**)	0.80	39 ± 7.4 ^4^	0.026	100
Diosmetin (**3**)	0.31	19.7 ± 1.73	0.016	60.2
5,7-Dihydroxy-3′,4′,5′-trimethoxyflavone (**4**)	0.27	30.4 ± 5.20	0.009	34.0
Jaceosidin (**7**)	0.77	43.5 ± 1.31	0.018	69.2
6-Methoxytricin (**8**)	0.16	23.2 ± 3.17	0.007	26.9
Eupatilin (**6**)	0.27	35.6 ± 2.52	0.008	29.0

^1^ The content of each compound in the CIC Fraction A was measured using HPLC and is shown as the percentage of dry weight of Fraction A. ^2^ Each value (mean ± SD) was calculated by at least three independent experiments using rat hepatocytes. ^3^ Content/IC_50_ ratio was set to 100% of the luteolin content/IC_50_ ratio. ^4^ Data from Nakajima et al. [14]. ND, not determined.

## Data Availability

The data presented in this study are available on request from the corresponding author.
